# Flumequine-Mediated Upregulation of p38 MAPK and JNK Results in Melanogenesis in B16F10 Cells and Zebrafish Larvae

**DOI:** 10.3390/biom9100596

**Published:** 2019-10-11

**Authors:** Wisurumuni Arachchilage Hasitha Maduranga Karunarathne, Ilandarage Menu Neelaka Molagoda, Myung Sook Kim, Yung Hyun Choi, Matan Oren, Eui Kyun Park, Gi-Young Kim

**Affiliations:** 1 Department of Marine Life Sciences, Jeju National University, Jeju 63243, Korea; hasikarunarathne@gmail.com (W.A.H.M.K.); neelakagm2012@gmail.com (I.M.N.M.); 2 Department of Biology, Jeju National University, Jeju 63243, Korea; myungskim@jejunu.ac.kr; 3 Department of Biochemistry, College of Oriental Medicine, Dong-Eui University, Busan 47227, Korea; choiyh@deu.ac.kr; 4 Department of Molecular Biology, Ariel University, Science Park, Ariel 40700, Israel; matanok@gmail.com; 5 Department of Oral Pathology and Regenerative Medicine, School of Dentistry, IHBR, Kyungpook National University, Daegu 41940, Korea; epark@knu.ac.kr

**Keywords:** flumequine, melanogenesis, mitogen-activated protein kinase

## Abstract

Flumequine is a well-known second generation quinolone antibiotic that induces phototoxicity. However, the effect of flumequine on skin melanogenesis is unclear. Therefore, we, for the first time, investigated whether flumequine regulates melanogenesis. The present study showed that flumequine slightly inhibited *in vitro* mushroom tyrosinase activity but significantly increased extracellular and intracellular melanin content in B16F10 cells and promoted the expression of microphthalmia-associated transcription factor (MITF) and tyrosinase. Additionally, flumequine remarkably increased melanin pigmentation in zebrafish larvae without any toxicity. We also found that flumequine stimulated p38 mitogen-activated protein kinase (MAPK) and c-Jun N-terminal kinase (JNK) phosphorylation; inhibition of p38 MAPK and JNK resulted in significant downregulation of extracellular and intracellular melanin content in B16F10 cells and pigmentation of zebrafish larvae accompanied with suppression of MITF and tyrosinase expression, indicating that flumequine-mediated p38 and JNK promote melanogenesis *in vitro* and *in vivo*. According to the molecular docking prediction, flumequine targeted dual-specificity MAPK phosphatase 16 (DUSP16), which is a major negative regulator of p38 MAPK and JNK. Our findings demonstrate that flumequine induces an increase in melanin content in B16F10 cells and zebrafish larvae by activating p38 MAPK and JNK. These data show the potential of flumequine for use as an anti-vitiligo agent.

## 1. Introduction

Melanin is produced by melanocytes distributed in the basal layer of the epidermis, which is a key element of the skin, hair, and eye color [1]. Under normal physiological conditions, melanin exerts beneficially protective effects against harmful ultraviolet (UV) radiation; however, excessive melanin production (hyperpigmentation) causes dermatological disorders such as freckles, age spots, and melisma [2]. In this regard, many attempts to discover medicinal flavonoids, and their derivatives and analogues that inhibit melanin biogenesis (melanogenesis), have been made over several decades [3,4]. In contrast, an acquired chronic depigmentation disorder such as vitiligo results in the functional loss of melanocytes, which makes an individual more vulnerable to UV [5].

For several decades, tyrosinase has been spotlighted as a main rate-limiting enzyme in the biosynthesis of melanin; tyrosinase catalyzes the hydroxylation of L-tyrosine to 3,4-dihydroxyphenylalanine (L-DOPA), and the subsequent oxidation of DOPA to dopaquinone [6]. Consequently, dopaquinone combines with cysteine to form pheomelanin and is alternatively converted to leucodopachrome and eumelanin [7]. Additionally, microphthalmia-associated transcription factor (MITF) is mainly involved in melanogenesis by activating cyclic adenosine monophosphate (cAMP)/protein kinase A (PKA), resulting in an increase in tyrosinase and tyrosinase-related protein expression [8,9]. In addition to cAMP/PKA axis activation by binding of α-melanocyte stimulating hormone (α-MSH) to its specific receptor, melanocortin 1 receptor (MC1R), mitogen-activated protein kinases (MAPKs) are particularly involved in regulating MITF expression [8]. In particular, data based on many depigmenting small-molecule compounds verified that extracellular signal-regulated kinase (ERK) downregulates melanogenesis through proteasomal degradation of MITF, thereby inhibiting melanogenesis; on the contrary, p38 MAPK and c-Jun N-terminal kinase (JNK) stimulates MITF-mediated tyrosinase activity, leading to hypermelanogenesis [10]. Even though some discrepancy has been found in the function of MAPKs during melanogenesis, MAPK regulation is a promising target for melanogenesis.

Dual-specificity MAPK phosphatases (DUSPs) are well established as negative regulators of MAPK signaling in mammals [11]. DUSPs are dedicated to their task by dephosphorylating both kinase interaction motif protein tyrosine phosphatases (KIM-PTPs) and serine/threonine protein phosphatase (PP2A) residues of the signature T-X-Y motif located within the active loop of the kinase [12]. DUSPs are divided into three subgroups based on sequence homology, substrate specificity, and subcellular localization: (1) DUSP1, DUSP2, DUSP4, and DUSP5 strongly induce ERK dephosphorylation and slightly inhibit p38 and JNK in the nucleus; (2) DUSP6, DUSP7, and DUSP9 are localized in the cytosol and selectively target ERK; and (3) DUSP8, DUSP10, and DUSP16 specifically inactivate p38 MAPK and JNK [13]. Nevertheless, whether DUSP-mediated MAPK regulation affects melanogenesis has not been elucidated.

Fluoroquinolones are orally deliverable antibiotics used to treat respiratory tract, gastrointestinal, and abdominal infections by targeting bacterial DNA gyrase (topoisomerase II (Topo II)) at the DNA replication stage [14,15]. Flumequine is a fluoroquinolone derivative applied in veterinary medicine for the treatment of enteric infections, but it is no longer used in the clinic [16]. Flumequine also targets bacterial DNA gyrase and blocks the replication of bacterial DNA, resulting in antibacterial activity and potency against gram-positive bacteria [17]. There is no evidence of the harmful effects of flumequine at low doses, but its excessive intake may be potentially risky in humans and animals [18]. Based on molecular docking analysis, Jadhav and Karuppayil showed that fluoroquinolones including flumequine form hydrogen bonds with active sites of human Topo IIa and Topo IIb, and could be a promising anti-cancer drug targeting Topo II [19]. However, the molecular action of flumequine on melanogenesis has not been understood.

In the current study, for the first time, we investigated the effect of flumequine on melanogenesis. Flumequine upregulated the activity and expression of melanogenic proteins such as tyrosinase and MITF by activating JNK and p38 MAPK, and molecular docking predicted that flumequine could interact with DUSP16.

## 2. Material and Methods

### 2.1. Regents and Antibodies

Dulbecco’s modified Eagle’s medium (DMEM), fetal bovine serum (FBS), and antibiotic mixtures were purchased from WELGENE (Gyeongsan-si, Gyeongsangbuk-do, Korea). Kojic acid, phenylthiourea (PTU), mushroom tyrosinase, 3-(4,5-dimethylthiazol-2-yl)-2,5-diphenyltetrazolium bromide (MTT), α-MSH, SB203580, and SP600125 were purchased from Sigma-Aldrich Co. (St. Louis, MO, USA). Antibodies against tyrosinase, MITF, JNK, phospho-JNK (p-JNK), p38, phospho-p38 (p-p38), and anti-β-actin were obtained from the Santa Cruz Biotechnology (Santa Cruz, CA, USA). Peroxidase-labelled anti-rabbit and anti-mouse immunoglobulins were obtained from KOMA Biotechnology (Seoul, Korea). All other chemicals were purchased from Sigma grades.

### 2.2. Cell Culture and Cell Viability Assay

B16F10 cells (ATCC, Manassas, VA, USA) were maintained in DMEM supplemented with 10% heat-inactivated FBS at 37 °C in a humidified atmosphere of 5% CO_2_. For cell viability, the MTT assay was performed. Briefly, B16F10 cells were seeded in 24 well plates at a density of 1 × 10^4^ cell/mL overnight at 37 °C. Then, the cells were treated with various concentrations (0–1000 μM) of flumequine for 72 h. After incubation, MTT was added to each well and incubated for 4 h at 37 °C. The precipitates were dissolved in DMSO (Sigma-Aldrich Co.), and absorbance was measured at 560 nm using a microplate spectrophotometer (Thermo Electron Corporation, Marietta, OH, USA). In a parallel experiment, cell morphology was analyzed under the CELENA^®^ S digital imaging system (Logos Biosystem, Gyeonggi-do, Korea).

### 2.3. Flow Cytometric Analysis

To estimate the total cell count, cell viability, and apoptotic cell population, flow cytometric analysis was carried out using Muse^®^ Count & Viability Assay Kit (Millipore, Billerica, MA, USA). In brief, B16F10 cells were plated at a density of 1 × 10^4^ cells/mL overnight and treated with the indicated concentrations of flumequine (0–400 μM) for 72 h. The cells were harvested and washed with ice cold phosphate-buffered saline (PBS). Then, the cells were incubated with the Muse^®^ Count & Viability Assay Kit for 5 min and analyzed according to the manufacturer’s instructions by Muse^®^ cellcycler (Millipore). H_2_O_2_ (100 μM) was used as an apoptosis-inducing control.

### 2.4. Cell Cycle Analysis

B16F10 cells from each group were washed three times and resuspended in 50 µL PBS. Suspended cells were added into the tube containing 1 mL of ice cold 70% ethanol in a dropwise manner while vortexing at gentle speeds. The tubes were frozen at −20 °C for 3 h prior to staining. Subsequently, the cells were washed and treated with 200 µl of Muse^®^ Cell Cycle reagent (Millipore) according to the manufacturer’s protocol. After 30 min of incubation at room temperature in the dark, cell suspension samples were transferred into 1.5 mL microcentrifuge tubes and analyzed using the Muse™ Cell Analyzer.

### 2.5. In Vitro Mushroom Tyrosinase Assay

The cell-free tyrosinase activity was measured by directly mixing mushroom and L-DOPA. Briefly, the reaction mixture was prepared with 130 µL of 100 mM phosphate buffer (pH 6.8), 20 µL of flumequine, 30 µL of 1.5 mM L-tyrosine, and 20 µL of 210 Units/mL mushroom tyrosinase, and then incubated for 30 min at 37 °C. Absorbance was measured at 490 nm. Kojic acid (25 µM) and PTU (250 nM) were used as positive controls.

### 2.6. Extracellular and Intracellular Melanin Content

B16F10 cells were cultured at 1 × 10^4^ cells/mL in 6-well plates overnight and treated with the indicated concentrations of flumequine (0–50 μM) for 72 h. α-MSH (500 ng/mL) was used as a positive control. Extracellular melanin content was measured using culture media at 405 nm. In order to measure intracellular melanin content, the cells were washed in ice-cold PBS and dissolved in 1 M NaOH containing 10% DMSO at 100 °C for 10 min. Then, absorbance was measured at 405 nm.

### 2.7. Reverse Transcription-Polymerase Chain Reaction (RT-PCR)

B16F10 cells were seeded at 1 × 10^4^ cells/mL in 6-well plates overnight at 37 °C. Then, the cells were treated with various concentrations of flumequine (0–50 μM) for 48 h, and α-MSH (500 ng/mL) was used as the positive control. The total RNA was extracted using the easy-BLUE™ total RNA extraction kit (iNtRON Biotechnology, Seongnam-si, Gyeonggi, Korea) following the manufacture’s protocol. The sequence of the sense and antisense primers for *Tyrosinase* were 5′-GTCGTCACCCTGAAAATCCTAACT-3′ and 5′-CATCGCATAAAACCTGATGGC-3′, respectively. *MITF* sense 5′-CCCGTCTCTGGAAACTTGATCG-3′, *MITF* antisense 5′-CTGTACTCTGAGCAGCAGGTC-3′, *Glyceraldehyde-3-phosphate dehydrogenase (GAPDH)* sense 5′-AGGTCGGTGTGAACGGATTTG-3′ and *GAPDH* antisense 5′-TGTAGACCATGTAGTTGAGGTCA-3′. The reaction sequence comprised 95 °C for 45 s, 62 °C for 45 s, and extended at 72 °C for 1 min for 25 cycles for each of *MITF* and *tyrosinase* and 94 °C for 30 s; and 60 °C for 30 s with and extension at 72 °C for 30 s for *GAPDH*. Agrose gel electrophoresis was performed to analyze the PCR products and visualized by ethidium bromide.

### 2.8. Western Blotting Analysis

B16F10 cells were seeded at 1 × 10^4^ cells/mL in 6-well plates overnight at 37 °C. Then, the cells were treated with the indicated concentrations of flumequine (0–50 μM) for 72 h. α-MSH (500 ng/mL) was used as a positive control. The cells were lysed with PRO-PREP lysis buffer (iNtRON Biotechnology). The supernatant was collected and its protein concentration was measured using Bio-Rad protein assay reagents (Bio-Rad, Hercules, CA, USA). The equal amount of protein was separated by electrophoresis on an SDS-polyacrylamide gel, transferred to a nitrocellulose membrane (Schleicher & Schuell, Keene, NH, USA), and then immunoblotted with the specific antibodies. Bound antibodies were detected using an enhanced chemiluminescence plus kit (Thermo Scientific, Rockford, IL, USA). The images were visualized by Chemi-Smart 2000 (Vilber Lourmat, Cedex, France). Images were captured using Chemi-Capt (Vilber Lourmat) and transported into Adobe Photoshop.

### 2.9. In Vivo Analysis of Melanogenesis in Zebrafish Larvae

AB strain zebrafishes were obtained from C.H. Kang (Nakdong National Institute of Biological Resources, Sangju, Gyeongsangbukdo, Korea) and cultured at 28.5 °C on a 14/10 h light/dark cycle. Embryos from natural spawning were collected in embryo medium (NaCl—34.8 g, KCl—1.6 g, CaCl_2_·2H_2_O—5.8 g, and MgCl_2_·6H_2_O—9.78 g with distilled water, pH 7.2) supplemented with 1% methylene blue at 28 °C. The chorion of the 1 day post-fertilized (dpf) zebrafish larvae was manually removed and pretreated with 200 µM PTU for 24 h (by 2 dpf). Then, the culture medium was replaced with flumequine (0–20 μM). α-MSH (1 µg/mL) was used as a positive control. Spontaneous melanin content was measured from zebrafish larvae at 5 dpf. After anesthetizing zebrafish larvae in tricane methane sulfonate solution at 5 dpf, the larvae were mounted in 2% methyl cellulose on a depression slide, and images were collected using an Olympus SZ2-ILST stereomicroscope (Tokyo, Japan). The densitometric analysis was performed using Image J software (National Institute of Health, Bethesda, MD). The quantification of pigmentation data was calculated as the percentage in comparison with the untreated control.

### 2.10. Analysis of the Heart Rate

The toxicity of flumequine was determined by measuring the heart rate of zebrafish at 5 dpf and compared to that of untreated controls. Counting of the heart rate was conducted with a camera under stereomicroscopy (Olympus SZ2-ILST). The obtained results were represented as average heart rate per minute.

### 2.11. Statistical Analysis

All the data in this study were obtained as an average of experiments that were performed at least in triplicate and expressed as mean ± standard error median (SEM). Statistical analysis was performed with Sigma plot 12.0 software using Student’s *t*-test and unpaired one-way analysis of variance (ANOVA) with Bonferroni correction. The significant significance of results was set at *p* < 0.05 (* and ^#^), *p* < 0.01 (** and ^##^), and *p* < 0.001 (*** and ^###^).

## 3. Results

### 3.1. Flumequine Slightly Downregulates Mushroom Tyrosinase Activity In Vitro

**Figure 1:**
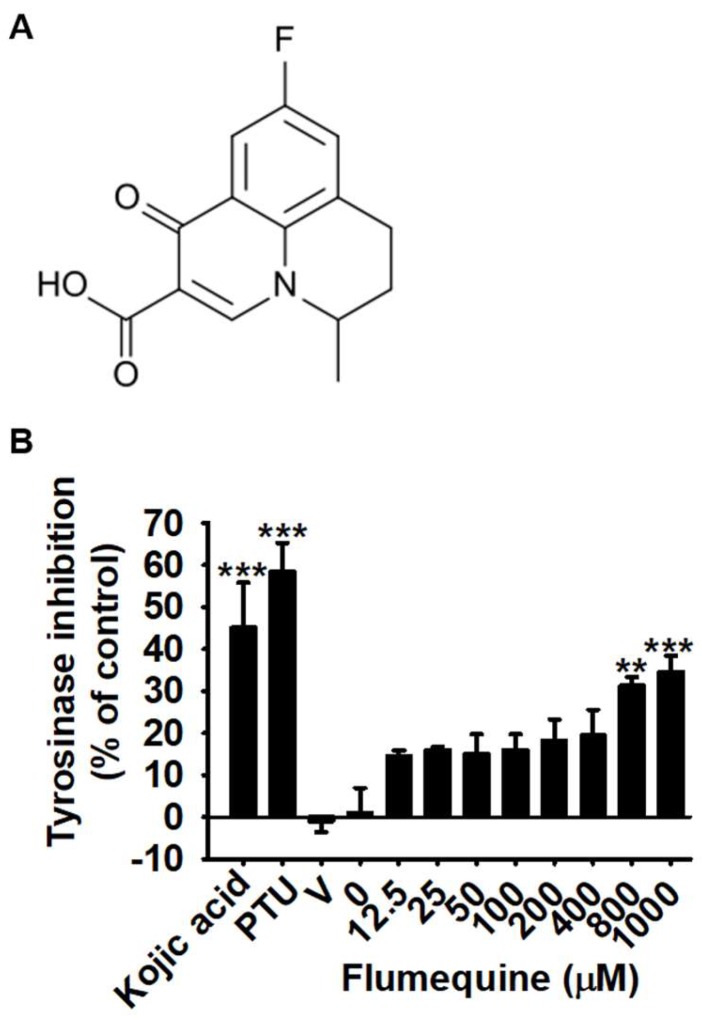
Flumequine slightly upregulates mushroom tyrosinase activity *in vitro* at high concentrations. (**A**) Chemical structure of flumequine. (**B**) The effect of flumequine on *in vitro* mushroom tyrosinase activity. Tyrosinase activity was determined by oxidation of L-DOPA as a substrate. Briefly, flumequine (0–1000 µM), kojic acid (25 µM), and phenylthiourea (PTU) (250 nM) were loaded into a 96-well microplate. After incubation with mushroom tyrosinase at 37 °C for 30 min, the dopaquinone level was measured by spectrophotometry at 490 nm. The results are the average of the three independent experiments and are represented as the mean ± standard error median (SEM). ***, *p* < 0.001 and **, *p* < 0.01 vs. untreated control. V, vehicle control (0.1% DMSO).

We first investigated whether flumequine (Figure 1A) positively or negatively regulates mushroom tyrosinase activity by quantifying the conversion of L-tyrosine to *O*-hydroxylated tyrosine and/or oxidation of l-DOPA to *O*-diquinone. As shown in Figure 1B, kojic acid and PTU are well-known tyrosinase inhibitors that both significantly increased the inhibition rate of mushroom tyrosinase by 45.1 ± 10.7% and 58.5 ± 6.8%, respectively. Flumequine showed no significant inhibitory effect on *in vitro* mushroom tyrosinase activity up to 400 µM compared to that in the untreated control. However, a 31.2 ± 2.1% and 34.6 ± 3.9% inhibition rate in tyrosinase activity was observed with 800 µM and 1000 µM flumequine, respectively. Additionally, molecular docking data showed that flumequine did not bind mushroom tyrosinase (PDB ID: 5M6B), indicating that low concentrations of flumequine did not directly inhibit tyrosinase activity *in vitro*; however, there is a possibility that high concentrations of flumequine non-specifically suppressed the tyrosinase reaction *in vitro*.

### 3.2. High Concentrations of Flumequine Slightly Decrease the Viability of B16F10 Cells, but Does Not Induce Cell Death and Arrest the Cell Cycle at S Phase

**Figure 2:**
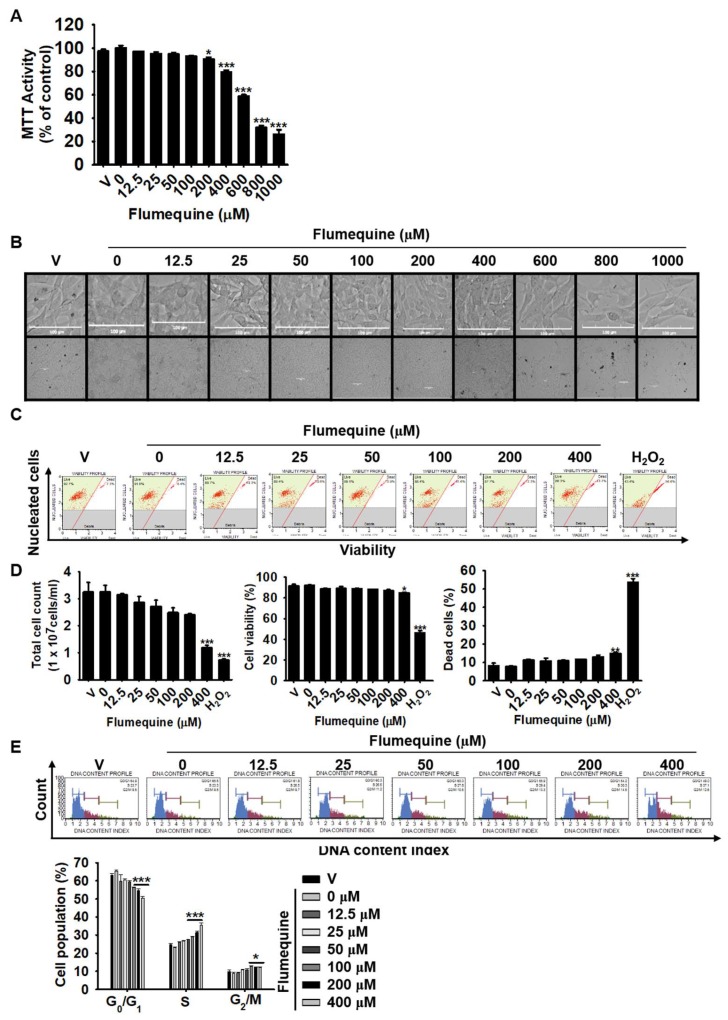
High concentrations of flumequine slightly decrease the viability of B16F10 cells. B16F10 cells were treated with 0–1000 µM flumequine for 72 h. (**A**) A 3-(4,5-dimethylthiazol-2-yl)-2,5-diphenyltetrazolium bromide (MTT) assay was performed to determine cell viability. Cell viability in each group was presented as the percentage of the values of the untreated control. (**B**) The cellular images were captured and analyzed. (**C**,**D**) B16F10 cells were treated with 0–400 µM flumequine for 72 h. Flow cytometric analysis was used to assess total cell count (left), cell viability (middle), and apoptotic cell population (right). (**E**) Cell cycle analysis carried out using the Muse™ cell cycle kit after treating with 0–400 µM flumequine for 72 h (top) and cell cycle distribution is represented (bottom). The results are the average of the three independent experiments; the data are expressed as the mean ± SEM: ***, *p* < 0.001, **, *p* < 0.01, and *, *p* < 0.05 vs. untreated control. V, vehicle control (0.1% DMSO).

To investigate the effect of flumequine on cell viability, B16F10 cells were treated with various concentrations (0–1000 µM) of flumequine for 72 h, and the MTT assay and microscopic analysis were performed. As shown in Figure 2A, a slight decrease in MTT activity was observed by 9.6 ± 1.7% at 200 µM flumequine in B16F10 cells, whereas MTT conversion activity was significantly decreased with 400 µM flumequine (21.8 ± 2.4%) and reached the lowest level at 1000 µM (73.9 ± 3.4%). However, no morphological change was seen at up to 400 µM flumequine, and a slight reduction in cell number was observed at over 600 µM under microscopic analysis (Figure 2B). Furthermore, flow cytometric analysis was performed to confirm the effect of flumequine on cell viability and cell death in detail (Figure 2C). As shown in Figure 2D, flumequine at 400 µM significantly reduced the total cell number ((1.8 ± 0.1) × 10^7^ cells/mL, left bottom); however, total cell viability was slightly decreased (14.9 ± 0.5%, middle bottom), and the dead cell population was slightly increased. Meanwhile, the apoptosis-inducing control H_2_O_2_ significantly increased dead cell population (54.7 ± 3.2%, right bottom). We next measured the cell cycle status of B16F10 cells in the presence of 0–400 µM flumequine at 72 h. Cell cycle distribution analysis showed that flumequine hampered the cell cycle progression by arresting the cells in S phase. According to Figure 2E, the cells in S phase were from 24.9 ± 0.6% (untreated control) to 35.6 ± 1.2% (400 µM flumequine) with a concomitant decrease in the percentage of cells in G_1_ phase from 63.1 ± 1.0% (untreated control) to 50.5 ± 0.9% (400 µM flumequine). Taken together, our data strongly suggest that high concentrations of flumequine (≥100 µM) does not induce apoptosis but causes an arrest of cells in S phase, and low concentrations of flumequine (≤ 50 µM) have no effect on cell death. Therefore, in all subsequent experiments, we used the lower range of concentrations.

### 3.3. Flumequine Increases Extracellular and Intracellular Melanin Production in B16F10 Cells

**Figure 3:**
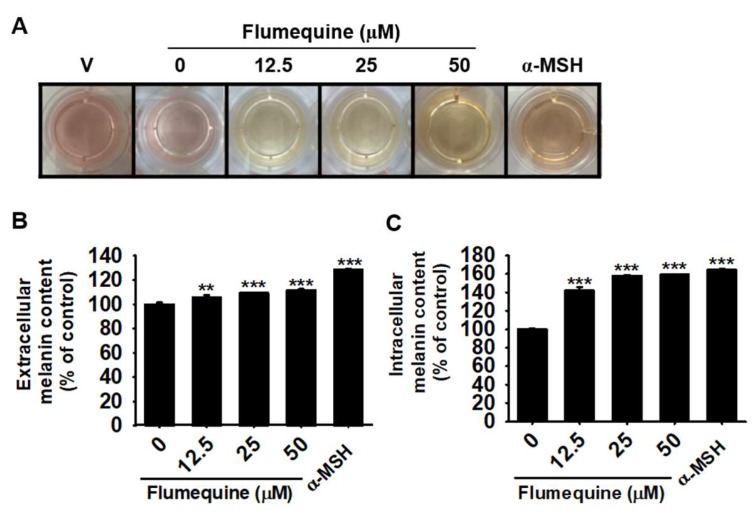
Flumequine increases the extracellular and intracellular melanin production in B16F10 cells. B16F10 cells were exposed to flumequine (0–50 µM) for 72 h. (**A**) Images of the culture medium color were captured. Extracellular (**B**) and intracellular (**C**) melanin content was measured at 72 h. The percentage values in each group are relative to those in the untreated control. The results are the average of three independent experiments and are represented as the mean ± SEM. ***, *p* < 0.001 and **, *p* < 0.01 vs. untreated control. V, vehicle control (0.1% DMSO).

To identify the effect of flumequine on melanogenesis, B16F10 cells were treated with various concentrations of flumequine (0–50 µM) for 72 h, and extracellular and intracellular melanin production was measured. As shown in Figure 3A, flumequine increased the brownish color in a dose-dependent manner compared to that of the untreated control. Based on the absorbance of supernatants at 405 nm, extracellular melanin content was increased by 6.1 ± 1.6%, 9.4 ± 0.5%, 11.6 ± 0.3% at 12.5 µM, 25 µM, and 50 µM flumequine, respectively, compared to that of untreated control (Figure 3B). Intracellular melanin content was also increased by 42.2 ± 3.6%, 58.1 ± 1.0%, 59.1 ± 0.6% at 12.5 µM, 25 µM, and 50 µM flumequine, respectively (Figure 3C). α-MSH used as a positive control strongly increased the extracellular (22.6 ± 2.4%) and intracellular melanin (64.6 ± 1.1%). These data indicate that flumequine stimulates melanogenesis in B16F10 cells.

### 3.4. Flumequine Stimulates Expression of MITF and Tyrosinase in B16F10 Cells

**Figure 4:**
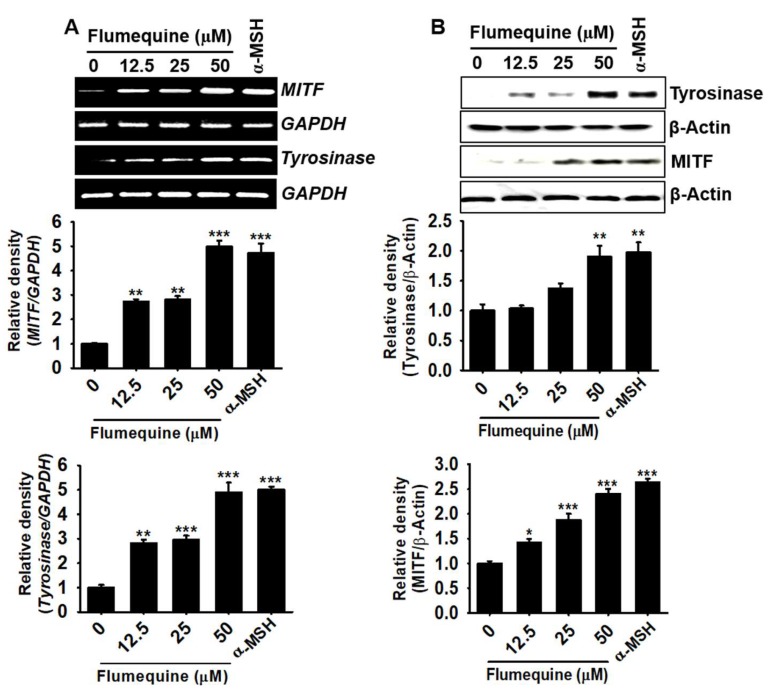
Flumequine stimulates microphthalmia-associated transcription (MITF) and tyrosinase expression in B16F10 cells. (**A**) B16F10 cells were exposed to flumequine (0–50 µM) for 48 h and the expression of *MITF* and *tyrosinase* was measured. (**B**) Under the same experimental condition, the protein expression of MITF and tyrosinase was measured by western blotting analysis at 72 h. The α-melanocyte stimulating hormone (α-MSH)-treated group was used as a positive control. The results are the average of three independent experiments and are represented as the mean ± SEM. ***, *p* < 0.001, **, *p* < 0.01, and *, *p* < 0.05 vs. untreated control.

To investigate the effect of flumequine on MITF and tyrosinase expression, RT-PCR and western blotting analysis were performed. As shown in Figure 4, flumequine increased both MITF and tyrosinase expression at the transcriptional level at 48 h (Figure 4A) and the translational level at 72 h in a dose-dependent manner (Figure 4B) compared to those in the untreated control. The highest concentration of flumequine (50 µM) significantly enhanced the expression of MITF and tyrosinase compared to that in the α-MSH-treated group. These data indicate that flumequine increases melanogenesis by inducing the expression of MITF and tyrosinase.

### 3.5. Flumequine Upregulates Melanin Pigmentation of Zebrafish Larvae

**Figure 5:**
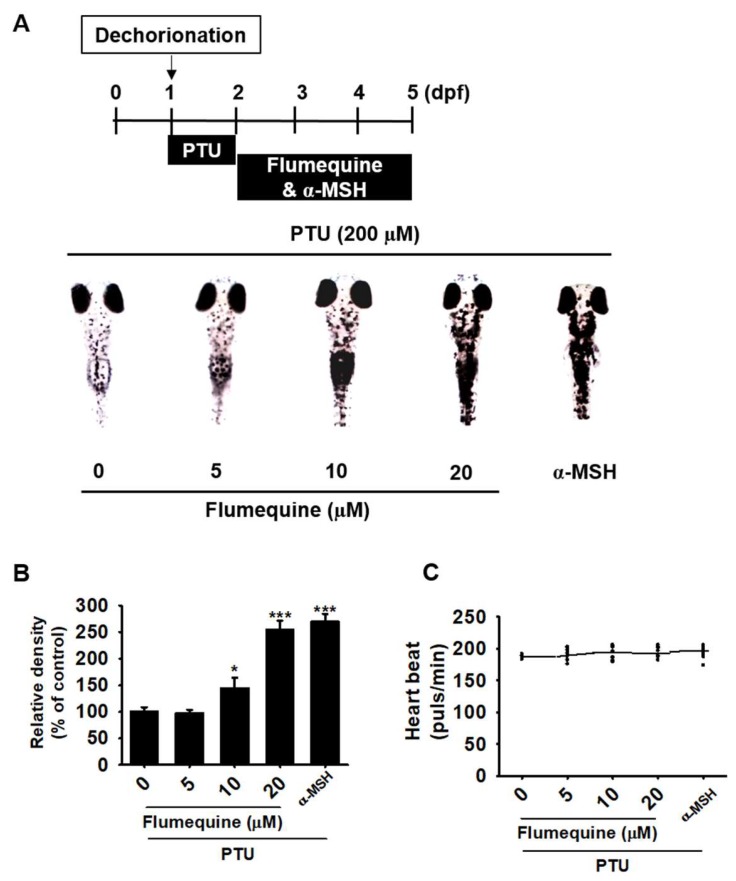
Flumequine upregulates melanin pigmentation in zebrafish larvae. (**A**) The chorion of zebrafish larvae (*n* = 20) was removed at 1 dpf and they were treated with PTU (200 µM) for 24 h (by 2 dpf). Then, flumequine (0–20 µM) was added to 2 dpf zebrafish larvae for 72 h (by 5 dpf), and images were captured at 5 dpf under an Olympus microscope (40×). (**B**) Relative density was calculated using Image J software. (**C**) Average heart beat of zebrafish larvae (*n* = 20) was measured to assess the toxicity of flumequine. The results are the average of three independent experiments and are represented as the mean ± SEM. ***, *p* < 0.001 and *, *p* < 0.05 vs. untreated control.

To further assess whether flumequine increases melanogenesis *in vivo*, zebrafish larvae were treated with flumequine, and melanin pigmentation and heart rate were measured. Consistent with hyperpigmentation in B16F10 cells, flumequine significantly increased melanin production in 5 dpf zebrafish larvae in a concentration-dependent manner (Figure 5A). Flumequine at 20 µM enhanced melanin pigmentation by as much as 2.5-fold (254.2 ± 17.3%) compared to that in the untreated control, which was comparable to that of the α-MSH-treated group (269.7 ± 15.1%) (Figure 5B). In addition, the zebrafish in all experimental groups showed the heart beat similar to that in the untreated control (192.4 ± 3.0) (Figure 5C). These results indicate that flumequine increases hyperpigmentation in vivo with non-toxicity.

### 3.6. Flumequine Induces p38 MAPK and JNK Phosphorylation, Resulting in Hypermelanogenesis

**Figure 6:**
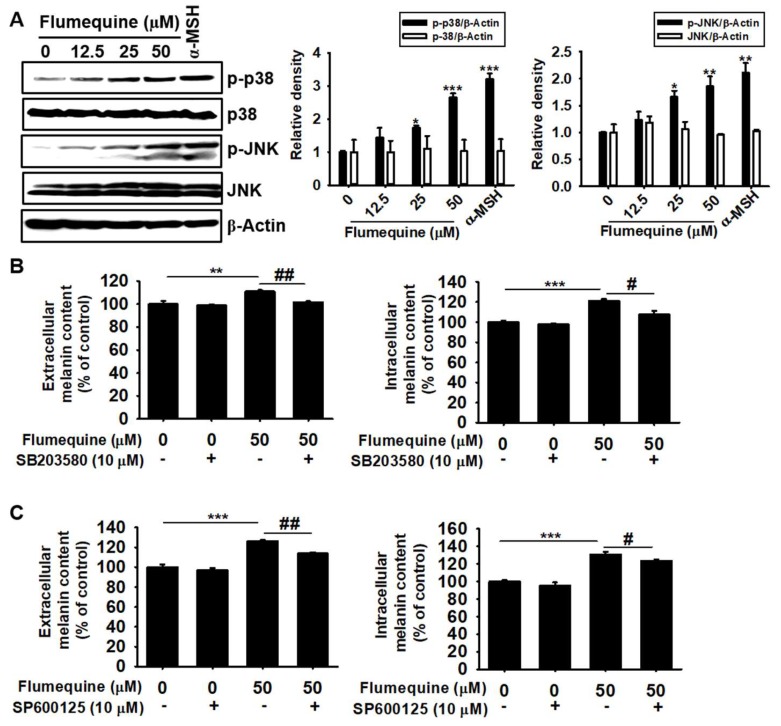
Flumequine induces p38 mitogen-activated protein kinase (MAPK) and c-Jun N-terminal kinase (JNK) phosphorylation, resulting in hypermelanogenesis. (**A**) B16F10 cells were exposed to flumequine (0–50 µM), and phosphorylation of p38 and JNK was measured by western blotting analysis at 72 h. In addition, B16F10 cells were pretreated with 10 μM SB203580 (**B**) or 10 μM SP600125 (**C**) for 1 h and were then treated with 50 µM flumequine for 72 h. Extracellular (left) and intracellular (right) melanin content was measured. The results are the average of three independent experiments and are represented as the mean ± SEM: ***, *p* < 0.001, **, *p* < 0.01, and *, *p* < 0.05 vs. untreated control, and ^##^, *p* < 0.01 and ^#^, *p* < 0.05 vs. flumequine-treated group.

Recent investigations indicate that phosphorylation of p38 MAPK and JNK induces melanogenesis by stabilizing MITF activation, which activates tyrosinase [8,19]. Therefore, we examined whether flumequine influences p38 and JNK activation. As shown in Figure 6A, flumequine induced p38 and JNK phosphorylation in a dose-dependent manner. For the functional activity of p38 MAPK and JNK on hypermelanogenesis, we examined the extracellular and intracellular melanin content in the presence of specific inhibitors of p38 (SB203580) and JNK (SP600125). As shown in Figure 6B,C, SB203580 and SP600125 significantly attenuated flumequine-induced upregulation of extracellular (left panel) and intracellular (right panel) melanin content in B16F10 cells. These results indicate that flumequine-mediated hypermelanogenesis is positively regulated by the p38 MAPK and JNK signaling pathway.

### 3.7. The p38 MAPK and JNK Signaling Pathway Upregulates Flumequine-Mediated Melanogenesis in B16F10 Cells and Zebrafish Larvae by Activating MITF and Tyrosinase

**Figure 7:**
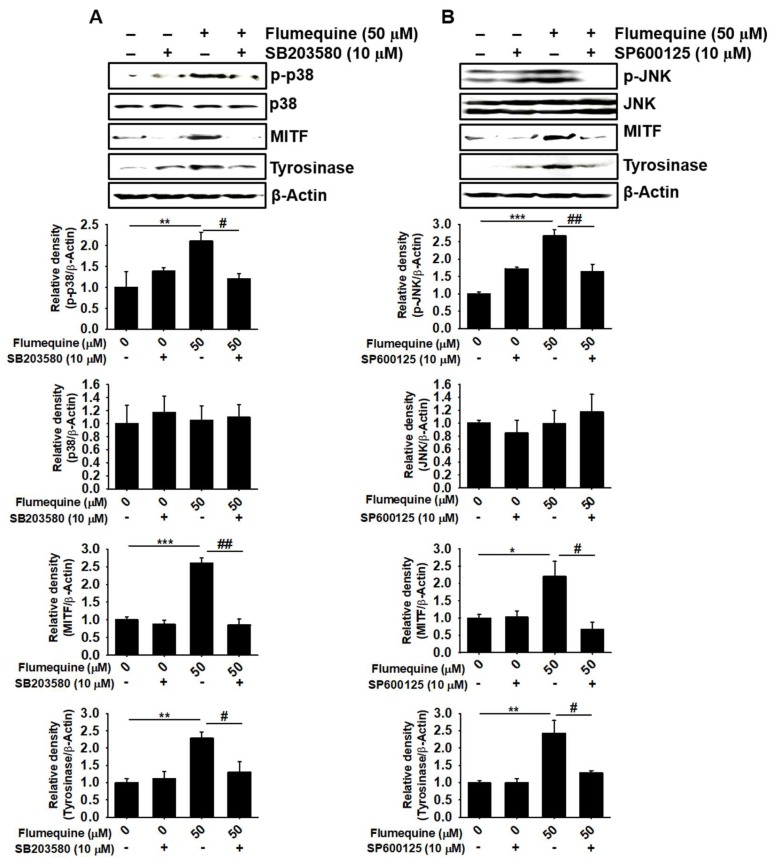
p38 MAPK and JNK upregulate flumequine-mediated melanogenesis in B16F10 cells by activating MITF and tyrosinase. B16F10 cells were pretreated with (**A**) SB203580 (10 μM) or (**B**) SP600125 (10 μM) for 1 h, and then flumequine (50 µM) was treated for 72 h. Expression of p38, JNK, MITF, and tyrosinase was measured by western blotting analysis. Each relative density was normalized by the density of β-Actin. The results are the average of three independent experiments and are represented as the mean ± SEM. ***, *p* < 0.001, **, *p* < 0.01, and *, *p* < 0.05 vs. untreated control, and ^##^, *p* < 0.01, ^#^, *p* < 0.05 vs. flumequine-treated group.

**Figure 8:**
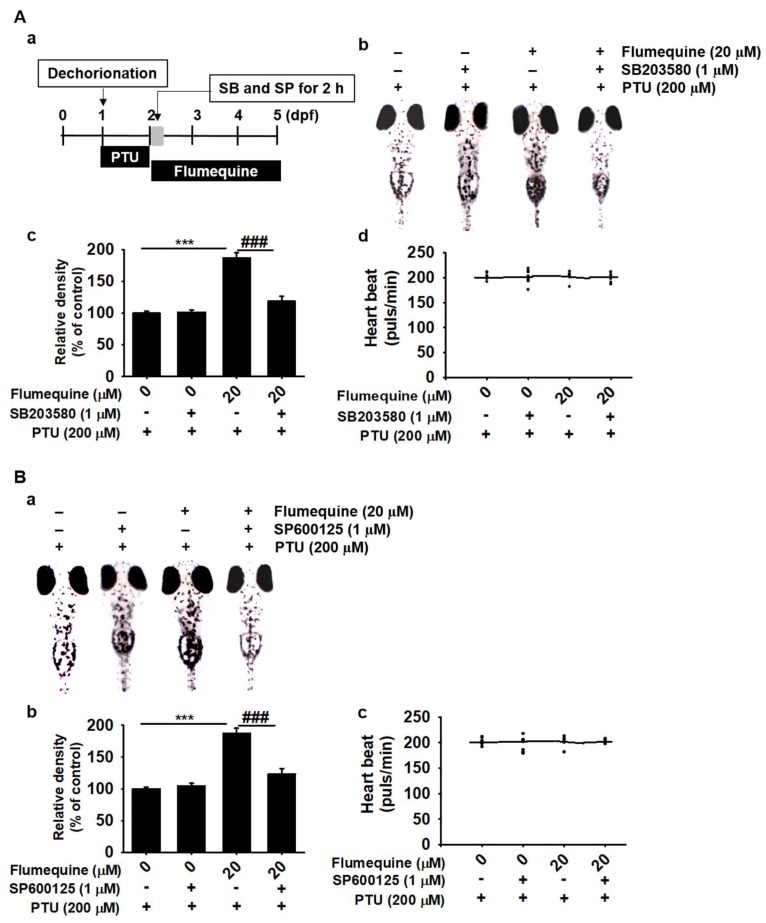
p38 MAPK and JNK upregulate melanogenesis in zebrafish larvae. (**A**-**a**) The chorion of zebrafish larvae (*n* = 20) was removed at 1 dpf and treated with PTU (200 µM) for 24 h. Then, 2 dpf zebrafish larvae were treated with flumequine (20 µM) for 72 h followed by treatment with SB203580 (1 μM) or SP600125 (1 μM) for 2 h. (**A**-**b**,**B**-**a**) Images of 5 dpf zebrafish larvae were captured under a microscope (40×). (**A**-**c**,**B**-**b**) Relative density was calculated using the Image J software. (**A**-**d**,**B**-**c**) The average heart rate of zebrafish larvae (*n* = 20) was measured to assess the toxicity of flumequine. Data are reported as the mean ± SEM of three independent experiments (*n* = 3). ***, *p* < 0.001 vs. untreated control and ^###^, *p* < 0.001 vs. flumequine-treated group preincubated with PTU.

To further elucidate the mechanism underlying the hypermelanogenic effect of flumequine, we examined the influence of a p38 MAPK inhibitor (SB203580) and a JNK inhibitor (SP600125) on the expression of MITF and tyrosinase. As shown in Figure 7A,B, flumequine increased MITF and tyrosinase expression along with p38 and JNK phosphorylation; however, SB203580 and SP600125 reversed the flumequine-induced increase in MITF and tyrosinase accompanied by p38 MAPK and JNK dephosphorylation. To confirm whether the downregulation of flumequine-mediated hypermelanogenesis in the presence of SB203580 and SP600125 is due to cytotoxicity, annexin V staining was measured using flow cytometry (Figure S1A). No apoptotic death was seen in the combined treatment with flumequine and SB203580 or SP600125 compared to that of the H_2_O_2_-treated group (Figure S1B). We further evaluated the effect of SB203580 and SP600125 on melanogenesis in zebrafish larvae (Figure 8A-a). Melanin pigmentation of zebrafish larvae was significantly increased in response to flumequine (20 µM) as much as 2-fold compared to that of the untreated control; however, SB203580 (Figure 8A-b,A-c) and SP600125 (Figure 8B-a,B-b) attenuated flumequine-induced pigmentation (192.8 ± 7.2% and 183.2 ± 8.1%, respectively) by 102.6 ± 5.4% and 118.8 ± 8.2%, respectively. In addition, flumequine showed no significant effect on the heart rate of zebrafish larvae and did not exhibit any morphological changes (Figure 8A-d,B-c). These results imply that flumequine enhances melanogenesis in vivo by activating p38 MAPK and JNK.

## 4. Discussion

Flumequine is known as a synthetic derivative of fluoroquinolone for the treatment of a wide range of bacterial infections including skin, ocular, urinary, respiratory, and gastrointestinal infections [16]. However, the use of flumequine has been partially limited in clinics, since ocular side effects were found in three patients treated with flumequine for urinary infection [20]. Additionally, many previous studies showed that fluoroquinolones have phototoxic reactions as one of the main side effects specifically in skin lesions with different degrees of severity and toxic epidermal necrolysis through unknown molecular mechanisms [21,22]. Recently, Beberok et al. reported that fluoroquinolones directly inhibit mushroom tyrosinase activity *in vitro* and intracellular melanin content in human melanocytes with a different phototoxic content, especially greater than that at 500 µM [23,24]; however, there is no report on whether flumequine also regulates melanogenesis. Interestingly, we found that flumequine increased melanin production in B16F10 cells and zebrafish larvae by activating the p38 MAPK and JNK signaling pathway, not through direct binding to tyrosinase.

Under UV irradiation, epidermal skin cells such as keratinocytes, melanocytes, and Langerhans cells produce α-MSH, which binds to MC1R on melanocytes, causing melanin production as a protective response against UV-mediated damage [25]. Binding of α-MSH to MC1R stimulates cAMP-mediated MITF activation, which binds to E-box sequences in the promoter regions of *tyrosinase* [26]. Recently, MAPKs were found to directly regulate the transcriptional activity of MITF and consequent MITF-mediated melanogenesis, but the functional role of MAPKs in melanogenesis is still controversial. Nevertheless, substantial data have confirmed that ERK inhibits tyrosinase expression through proteasome-mediated degradation of MITF, leading to anti-melanogenesis; in contrast, melanogenesis is strongly induced by the activation of p38 MAPK and JNK, which stimulate MITF-mediated tyrosinase expression [10]. Our results showed that flumequine significantly activated p38 and JNK phosphorylation and that inhibitors of p38 (SB203580) and JNK (SP600125) inhibited melanogenesis along with downregulation of MITF and tyrosinase in B16F10 cells and zebrafish larvae, indicating that flumequine promotes melanogenesis via an increase in p38 and JNK phosphorylation. Some small flavonoids are known to stimulate melanogenesis by activating ERK [27,28], but p38-mediated anti-melanogenesis was also reported [29]. We still do not know whether MAPK family members play a dual role in melanogenesis depending on stimulants or small chemicals such as flavonoids. To determine the function of MAPKs in melanogenesis, further studies using different experimental models are needed.

Unexpectedly, molecular docking analysis showed no direct binding of flumequine with tyrosinase. Therefore, we focused on DUSPs, a family of proteins that function as key negative regulators of MAPKs in mammalian cells [11]. DUSPs are generally categorized into three subgroups depending on the amino acid sequence homology, subcellular localization, and substrate specificity. DUSP1, DUSP2, DUSP4, and DUSP5 are inducible nuclear phosphatases targeting ERK dephosphorylation; DUSP6, DUSP7, and DUSP9 are cytoplasmic ERK-specific phosphatases; and DUSP8, DUSP10, and DUSP16 are present in both the cytoplasm and cell nucleus and are relatively selective in their ability to dephosphorylate p38 MAPK and JNK [13]. Interestingly, molecular docking data showed that the dimer DUSP16 (PDB: 2VSW) binds flumequine in four predicted docking poses with no significant hydrogen bonds (Figure S2A, one of the strongest binding activities of flumequine to dimer DUSP16). Docking scores were −1.8, −1.5, −1.2, and −1.0, respectively (Table S1). Tetramer DUSP16 (PDB: 3TG3) also showed four different binding predictions with flumequine. Figure S2B shows the predicted pose 1 that has the strongest binding activity. In particular, predicted poses 1, 2, and 3 showed that flumequine interacts via SER82, SER117, and SER117 at distances of 2.066Å, 3.261Å, and 3.268Å, respectively (docking score: −3.9, −3.9, and −3.8, respectively) (Figure S2C and Table S1), suggesting that flumequine could bind to DUSP16 and inhibit it, resulting in the activation of p38 MAPK and JNK. Additionally, we predicted the molecular docking of DUSP8 (PDB: 4JMK) and DUSP10 (PDB: 2OUC) with flumequine but found that flumequine did not interact with DUSP8 and DUSP10. The data reveal that flumequine specifically interacts with DUSP16 and consequently stimulates hypermelanogenesis by activating p38 MAPK and JNK.

## 5. Conclusions

In conclusion, our study demonstrates that flumequine increases the melanin content in B16F10 melanoma cells and zebrafish larvae by activating p38 MAPK and JNK. These results suggest that flumequine could be used as an anti-vitiligo agent because low concentrations of flumequine stimulate melanin production.

## Supplementary Materials

The following are available online at https://www.mdpi.com/2218-273X/9/10/596/s1, Figure S1: Flumequine does not induce apoptosis in the presence of SB203580 or SP600125, Figure S2: Molecular docking comparison of flumequine with DUSP16, Table S1: Classification of results gained from the docking of flumequine into DUSP16.
