# Supplementary material for: Flumequine-Mediated Upregulation of p38 MAPK and JNK Results in Melanogenesis in B16F10 Cells and Zebrafish Larvae
Source: Biomolecules. 2019 Oct 11;9(10):596. doi: 10.3390/biom9100596 (PMC6843389; doi:10.3390/biom9100596)
Supplement: Supplementary file 1 [file biomolecules-09-00596-s001.pdf]

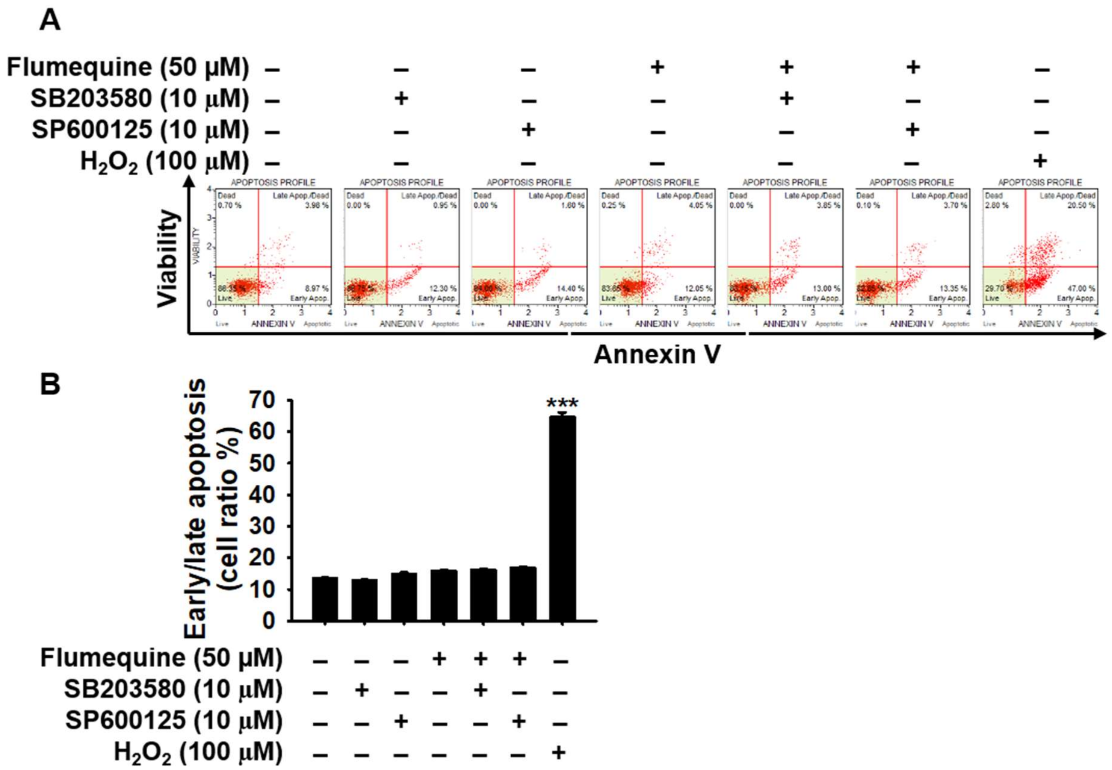

Figure S1. Flumequine does not induce apoptosis in the presence of SB203580 or SP600125. B16F10 cells were seeded overnight, treated with SB203580 (10 μM) or (B) SP600125 (10 μM) for 1 h, and then treated with flumequine (50 μM) for 72 h. (A) The various stage of apoptosis was measured by the Muse™ Annexin V and Dead Cell assay. Early and late apoptosis was shown in right bottom and right top. (B) Total percentage of apoptosis (early/late apoptosis) was represented. H_2O_2 was used as an apoptosis-inducing positive control. ***, p < 0.001 *vs*. untreated control.

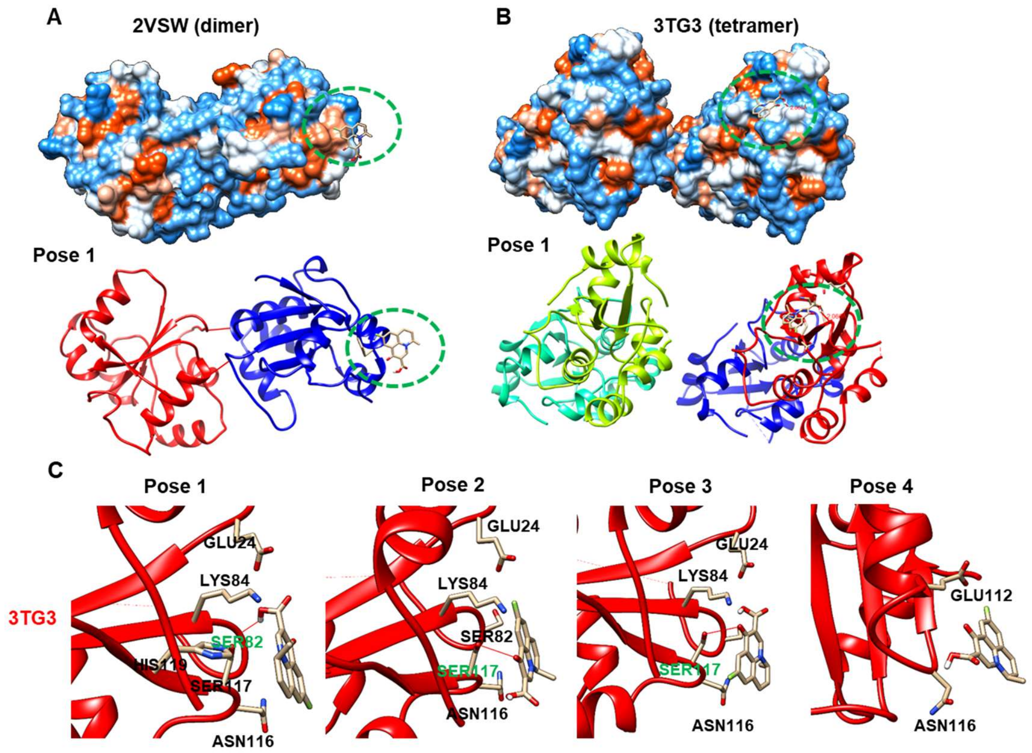

Figure S2. Molecular docking comparison of flumequine with DUSP16. (A) Surface structure of dimer DUSP16 (PDB: 2VSW) and (B) tetramer DUSP16 (PDB: 3TG3) binds to flumequine (top). Ribbon shape represents pose 1-binding activity, which is highest docking score (bottom). (C) Four different docking poses of tetramer DUSP16 (PDB: 3TG3) were presented.

Table S1. Classification of results gained from the docking of flumequine into DUSP16.

Receptor	Docking pose	Docking score	Binding A.A.* (H-bond)**	H-bond distance (Å)
DUSP16 (2VSW)	1	-1.8	N.F.	N.D.
	2	-1.5	N.F.	N.D.
	3	-1.2	N.F.	N.D.
	4	-1.0	N.F.	N.D.
DUSP16 (3TG3)	1	-3.9	SER82	2.066
	2	-3.9	SER117	3.261
	3	-3.8	SER117	3.268
	4	-3.6	N.F.	N.D.

* A.A.: amino acid

** H-bond: hydrogen bond

N.F.: not found

N.D.: not determined
